# Prevalence and recurrence of bacteraemia in hospitalised people who inject drugs – a single Centre retrospective cohort study in Denmark

**DOI:** 10.1186/s12879-020-05357-0

**Published:** 2020-08-26

**Authors:** Sara Thønnings, Filip Jansåker, Christoffer Sundqvist, Rebekka Faber Thudium, Susanne Dam Nielsen, Jenny Dahl Knudsen

**Affiliations:** 1 Department of Virus & Microbiological Special Diagnostics, Statens Serum Institute, Copenhagen, Denmark; 2 Department of Clinical Microbiology, Copenhagen University Hospital, Rigshospitalet, Copenhagen, Denmark; 3 Center for Primary Health Care Research, Lund University, Malmö, Sweden; 4 Department of Clinical Microbiology, Copenhagen University Hospital, Hvidovre Hospital, Hvidovre, Denmark; 5 Department of Infectious Diseases, Copenhagen University Hospital, Rigshospitalet, Copenhagen, Denmark; 6 Department of Infectious Diseases, Copenhagen University Hospital, Hvidovre Hospital, Hvidovre, Denmark

**Keywords:** PWID, Bacteraemia, Recurrence, Mortality, *S. aureus*

## Abstract

**Background:**

People who inject drugs (PWID) have increased risk of acquiring blood-transmitted chronic viral infections such as Hepatitis B (HBV), Hepatitis C (HCV) and Human Immunodeficiency Virus (HIV) as well as increased risk of acquiring bacterial infections. We aimed to identify and describe bacteraemic episodes, their recurrence rates, predictive and prognostic factors amongst hospitalised PWID.

**Methods:**

In this retrospective cohort study, we included 257 hospitalised PWID during 2000–2006 with follow up at the Department of Infectious Diseases, Hvidovre Hospital, Denmark. Data collection included comorbidity (HBV-, HCV-, HIV-, and psychiatric comorbidities), social information (contact to an addiction treatment centre, homelessness), opioid substitution treatment (OST), treatment completion and microbiology findings. There was a 10-years follow-up regarding mortality.

**Results:**

The study identified 257 patients classified as PWID. Of these, 58 (22.6%) had at least one episode of bacteraemia during their first hospital admission. Recurrence was found in 29 (50.0%) of the bacteraemia cases. *Staphylococcus aureus* was the dominant microorganism of both first and recurrent episodes with 24 (41.4%) and nine (31.4%) of cases, respectively. A psychiatric diagnose was significantly associated with a lower risk of bacteraemia in the multivariate analysis (OR: 0.29, [95%CI: 0.11–0.77], *P* = 0.01). Mortality was significantly higher in patients with bacteraemia (17.2% vs. 3.0%, *P* < 0.01, OR: 6.67 [95%CI: 2.33–20], *P* < 0.01).

**Conclusions:**

In hospitalised PWID, bacteraemia was found in 22.6% and was associated with at higher mortality. The most common microorganism of bacteraemia was *S. aureus*. Psychiatric comorbidity was significantly associated with a lower risk of bacteraemia.

## Background

Denmark has a population of approximately 5.7 million residents, and it is estimated that 33,074 people (5.8/1000 inhabitants) have persistent usage of illicit drugs. About 13,000 (2.3/1000 inhabitants) of these are people who inject drugs (PWID) [1].

Mortality among PWID is known to be much higher than in the general population, and bacterial infections are common causes for hospitalisations and death [2]. As a result of intravenous (IV) drug administration, most bacterial infections are caused by the subjects’ own commensal flora, with *Staphylococcus aureus* being the most common cause of bacteraemia as well as skin- and soft-tissue infections [3, 4]. Data regarding bacteraemia in PWID in Denmark is sparse, yet previous studies indicate a higher prevalence of bacteraemia in this patient category [5, 6]. Bacteraemia is a serious condition that strongly affects the patients morbidity and mortality, and bacteraemic episodes and their recurrence rates among the general population have previously been investigated in Denmark showing a recurrence rate of 12% within one year [7]. Compliance to treatment is a challenge for PWID, which may influence the recurrence rates of bacteraemia in this population. In addition, PWID have a high prevalence of chronic viral infections [8]. Hence, the aims of this study were to describe bacteraemic episodes and their recurrence rates in PWID admitted to the hospital over a six-year period. Moreover, we wanted to determine prognostic factors for bacteraemic episodes and their recurrence and study associations with mortality.

## Methods

### Setting and data sources

This retrospective cohort study included patients from 2000 to 2006 admitted at Copenhagen University Hospital, Hvidovre, Denmark. All Danish residents are given a unique 10-digit identification number used for all health care contacts [9]. Health care in Denmark is tax-supported and free of charge for all Danish residents, including substance abuse treatment [1].

Patients were identified retrospectively using a database search of hospital admissions to Copenhagen University Hospital, Hvidovre from 1st January 2000 to 31st December 2006. The following search-terms were used: dependence, chronic HCV infection, endocarditis, abscesses and osteomyelitis. Patients identified during the primary search were further screened through medical records. Inclusion criteria were age more than 18, hospital admission and drug abuse including injection of drugs. No exclusion criteria were applied. Further data regarding the patients included in the retrospective cohort were collected from medical records using a standardised data collection form. Data collected at the time of first hospital admission were: Contact to an addiction treatment center, homelessness, co-morbidities (i.e. HBV-, HCV-, HIV-, psychiatric comorbidities and use of psychiatric treatments), opioid substitution treatment (OST), and treatment completion. Furthermore, the dataset included microbiological findings between 1st January 2000 and 31st July 2008 which were retrieved from the local microbiology database. The cohort had a 10-year follow up regarding mortality. Supplementary Fig. 1 present the timeline of the study.

### Microbiology

A clinical physician did all microbiological samplings at clinical indications. Blood cultures were performed using two sets of one aerobic and one anaerobic bottle (BactAlert, bioMerieux, France). The positive blood cultures were sub-cultured, and the microorganisms were identified by standard methods [10], and susceptibility testing was performed by disc diffusion methods according to guidelines provided by EUCAST (www.eucast.org).

### Viral infections and bacteraemia

A clinical physician ordered all laboratory analyses. HBsAg, anti-HBs, anti-HCV and anti-HIV were analysed using Electrochemi-luminescens Immunoassay “ECLIA” (COBAS6000, Roche Diagnostics GmbH, Mannheim, Germany). HCV-RNA were analysed using real-time transcription-mediated amplification (Panther system, Hologic Inc., San Diego, CA).

HBV-status was classified according to HBsAg and anti-HBs. The presence of HBsAg was defined as acute or chronic infection and presence of anti-HBs was defined as resolved infection or due to immunization. HCV-status was classified according to detectable HCV-RNA and anti-HCV; Presence of only HCV-RNA was defined as an acute infection, presence of both HCV-RNA and anti-HCV as chronic infection and presence of only anti-HCV as resolved infection. HIV-status was recorded based on anti-HIV. A bacteraemic episode was defined as growth of any blood culture isolate within 48 h from admission to the hospital. Recurrent bacteraemia was defined as growth of any other blood culture isolate beyond 48 h from the first isolate from a new blood culture set, or the same microorganism more than 30 days after the initial bacteraemia within two years. Coagulase-negative staphylococci, *Corynebacterium species* and *Cutibacterium acnes* were classified as contaminants, unless they were isolated from two or more separate blood-culture sets. Fungaemia was for convenience also classified as bacteraemia.

### Medication, comorbidity and mortality

Any prescribed dose of methadone or buprenorphin at the time of admission was defined as OST. Psychiatric comorbidity was defined as any psychiatric diagnosis prior to hospital admission. Patients were registered as homeless when they had no residential address and were not residents on an institution. Contact to an addiction treatment center was defined as any present contact to an addiction treatment centre. Treatment was registered as completed if the patient followed the scheduled treatment plan (e.g. was not discharged prior to schedule treatment ending or failed to show in the outpatient clinic).

The mortality was defined as all-cause mortality from the day of hospital admission. In case of recurrent bacteraemia the latest admission with a positive blood culture was used to calculate all-cause mortality. Mortality was either 30-day mortality or 10-year mortality.

### Statistical analysis

Continuous data were presented as medians with interquartile ranges. Categorical data were analysed with the Fisher’s exact test with odds ratio (OR) presented with 95% confidence interval (95% CI). Univariate OR with 95% CI for bacteraemia and recurrence of bacteraemia were calculated using logistic regression. Variables with *P* < 0.30 were included in multivariate analyses for predictive factors of bacteraemia. Survival curves were presented as Kaplan-Meier curves and compared using the Log-rank test. No patients were excluded in the analysis. Two-sided significance was tested with the assumption of *P* < 0.05 as statistically significant.

The Statistical Package for Social Sciences (version 26.0; SPSS, IBM) was used for the analysis.

### Ethical considerations and data protection approval

The Danish Data Protection Agency approved the study (7–505–29-369/1).

## Results

### Baseline data

**Fig. 1:**
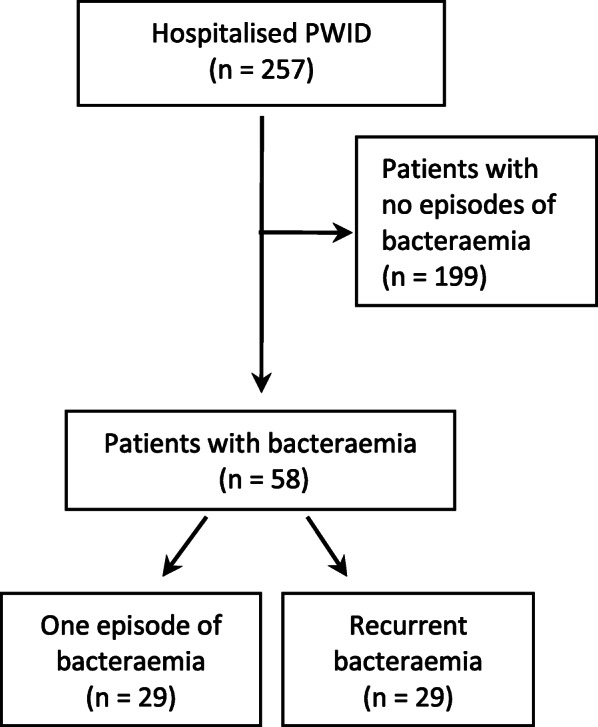
Flow diagram presenting the inclusion and exclusion procedure for hospitalised people who inject drugs (PWID) with bacteraemia and recurrent bacteraemia

Figure 1 shows a flow chart of the included patients. In the six-year study period from 2000 to 2006, we identified 257 hospitalised PWID. Acute or chronic HBV-infection were found in 4.3% of the patients and 19.5% had a resolved infection or were immunised (Table 1). Chronic hepatitis C was seen in 39.7% of all patients, and another 22.6% could not be distinguished from either chronic or resolved infection because of a missing data on HCV-RNA. Almost half of the patients were HIV-positive. Table 1 and Supplementary Table 1 shows the full patients status regarding, hepatitis B, hepatitis C and HIV.

**Table 1:** Clinical characteristics of 257 hospitalised people who inject drugs

Clinical characteristics:		PWID *n* = 257 (%)
Age (median) (IQR)		38 (34–45)
HBV-status	Acute/Chronic	11 (4.3)
	Resolved/Immunised	50 (19.5)
	Negative	79 (30.7)
	Unknown	117 (45.5)
HCV-status	Acute	1 (0.4)
	Chronic	102 (39.7)
	Resolved	12 (4.7)
	Chronic or resolved	58 (22.6)
	Negative	8 (3.1)
	Unknown	76 (29.6)
HIV-status	Positive	127 (49.4)
	Negative	101 (39.3)
	Unknown	29 (11.3)
HIV and HCV co-infection		88 (34.2)
Psychiatric comorbidity		53 (20.6)
Opioid substitution treatment		210 (81.7)
Homeless		74 (28.8)
Contact to an addiction treatment center		169 (65.8)

If not stated otherwise, results are presented as n (%)

*Abbreviations*: *PWID* people who inject drugs, *HIV* Human Immunodeficiency Virus, *HBV* hepatitis B, *HCV* hepatitis

In the cohort, 58 (22.6%) PWID had at least one episode of bacteraemia. Blood cultures from the remaining 199 patients was either negative or blood cultures were not performed. Recurrent bacteraemia was found in 29 patients, of whom 27 had recurrence within one year.

### Microbiological findings in first and recurrent bacteraemia

The first episode of bacteraemia was in 48 patients mono-microbial (82.8%). Mono-microbial Gram-positive bacteraemia accounted for 43 (74.1%) of all the first episodes (*n* = 58) and 22 (75.9%) of the recurrent episodes (*n* = 29).

*Staphylococcus aureus* accounted for 24 (41.4%) of all first episodes of bacteraemia and nine (31.0%) of recurrent episodes. *Streptococcus pneumoniae* was the second most common microorganism with six (10.3%) of all first episodes. Only one bacteraemic episode was caused by *Escherichia coli*.

Among the recurrent episodes, non-haemolytic streptococci were the second most common finding with three (10.3%) cases. Two episodes with yeasts were both in recurrent episodes. Of all the recurrent episodes, recurrence of the presumed same microorganism as in the first bacteraemic episode was only found for *S. aureus* in six out of ten episodes (see Supplementary Table 2).

### Predictors of bacteraemia and recurrence

Patients with bacteraemia (*n* = 58) were compared to patients without bacteraemia (*n* = 199) (Table 2). In univariate analysis psychiatric comorbidities were less common among patients with bacteraemia (8.6% vs. 24.1%, OR: 0.30 [95%CI: 0.11–0.76], *P* = 0.01). Adjusting for age, HBV, HCV and HIV in the multivariate analyses, psychiatric comorbidities were still significantly associated with a lower risk of bacteraemia (adjusted odds ratio (aOR): 0.29 [95%CI: 0.11–0.77], *P* = 0.01). Comparing patients with recurrent bacteraemia (*n* = 29) to patients without recurrence (*n* = 29) no significant predictive factors were found (Table 3).

**Table 2:** Predictive factors of bacteraemia in hospitalised people who inject drugs

Predictors	Total *n* = 257 (%)	Bacteraemia *n* = 58 (%)	Non-bacteraemia *n* = 199 *(*%)	Univariate analysis		Multivariate analysis *P* < 0.3	
				OR^A^ (95% CI^B^)	*P* value	Adjusted OR^A^ (95% CI^B^)	*P* value
Age (years)	39 (34–45)	40 (34–45)	39 (33–44)	1.02 (0.99–1.05)	0.27	3.46 (1.30–9.23)	0.21
HBV (acute or chronic)	11 (4.3)	4 (6.9)	7 (3.5)	2.03 (0.57–7.20)	0.27	1.42 (0.34–5.95)	0.63
HCV (acute or chronic)	103 (40.1)	17 (29.3)	85 (42.7)	0.56 (0.30–1.05)	0.07	0.57 (0.30–1.08)	0.08
HIV positive	127 (49.4)	25 (43.1)	102 (51.3)	0.72 (0.40–1.30)	0.30	0.72 (0.39–1.32)	0.29
Psychiatric comorbidity	53 (20.6)	5 (8.6)	48 (24.1)	0.30 (0.11–0.76)	**0.01**	0.29 (0.11–0.77)	**0.01**
Opioid substitution treatment	210 (81.7)	52 (89.7)	158 (79.4)	2.25 (0.90–5.60)	0.84		
Homeless	74 (28.8)	14 (24.1)	60 (30.2)	0.74 (0.38–1.45)	0.41		
Contact to an addiction treatment center	169 (65.8)	40 (69.0)	129 (64.8)	1.21 (0.64–2.26)	0.64		

^A^Odds Ratio (OR); ^B^Confidential Interval (CI). *P* < 0.05 in bold

If not stated otherwise, results are presented as n (%)

*Abbreviations*: *PWID* people who inject drugs, *HIV* Human Immunodeficiency Virus, *HBV* hepatitis B, *HCV* hepatitis C

**Table 3:** Predictive factors of recurrent bacteraemia in hospitalised people who inject drugs

Predictors	Total *n* = 58 (%)	Bacteraemia with recurrence *n* = 29 (%)	Non-recurrent bacteraemia *n* = 29 (%)	OR^A^ (95% CI^B^)	*P* value
Age (years)	40 (35–45)	39 (31–43)	40 (37–47)	0.93 (0.86–1.01)	0.10
HBV (acute or chronic)	4 (6.9)	1 (3.4)	3 (10.3)	0.31 (0.03–3.17)	0.61
HCV (acute or chronic)	17 (29.3)	6 (20.7)	11 (37.9)	0.43 (0.13–1.38)	0.25
HIV positive	24 (41.4)	12 (41.4)	12 (41.4)	0.87 (0.31–2.43)	1.00
Psychiatric comorbidity	5 (8.6)	1 (3.4)	4 (13.8)	0.22 (0.02–2.13)	0.35
Opioid substitution treatment	52 (89.7)	28 (96.6)	24 (82.8)	5.88 (0.64–50.00)	0.19
Homeless	14 (24.1)	5 (17.2)	9 (31.0)	2.16 (0.62–7.49)	0.36
Contact to an addiction treatment center	40 (69.0)	21 (72.4)	19 (65.5)	1.39 (0.45–4.17)	0.78
Treatment completed	17 (29.3)	11 (37.9)	6 (20.7)	2.48 (0.77–8.04)	0.16

^A^Odds Ratio (OR); ^B^Confidential Interval (CI)

If not stated otherwise, results are presented as n (%)

*Abbreviations*: *PWID* people who inject drugs, *HIV* Human Immunodeficiency Virus, *HBV* hepatitis B, *HCV* hepatitis C

### Mortality

**Fig. 2:**
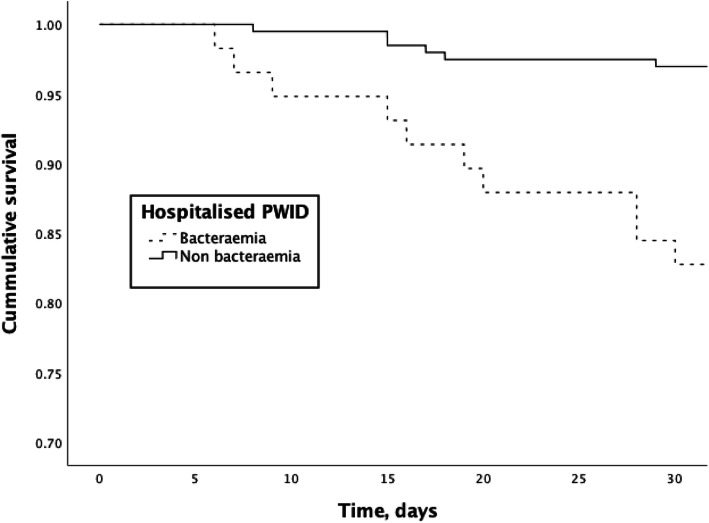
Survival curves showing mortality amongst hospitalised people who inject drugs stratified on bacteraemia. Log Rank Test *P* < 0.01 (Kaplan-Meier plot)

The 30-day mortality in the cohort was 6.2% (16/257). Patients with bacteraemia had a significantly higher mortality compared to non-bacteraemic patients both in univariate analysis (17.2% vs. 3.0%, OR: 6.67 [95%CI: 2.33–20], *P* < 0.01) and comparing the survival curves (Log-rank test *P* < 0.01, Fig. 2). Mortality was still significantly higher 10 years after (65.5% vs. 48.7%, OR: 2.00 [95%CI: 1.09–3.70], *P* = 0.03).

Subgroup analysis of the patients with bacteraemia showed no difference in 10-years mortality when comparing patients with recurrent bacteraemia vs. non-recurrent bacteraemia (65.5% vs. 65.5%, OR 1.00 CI 0.34–2.95, *P* = 1.00).

## Discussion

In this retrospective cohort study, we found that 22.6% of hospitalised PWID had at least one episode of bacteraemia during their hospital admission. Bacteraemia was associated with significantly higher mortality, which is in accordance with previous studies [11–14]. In addition, half of the cohort with bacteraemia had recurrent bacteraemia during the study period. Studies among the general population have reported recurrence rates of 9–12% [7, 15]. However, higher incidence of recurrence among PWID was expected. First, a higher incidence of overall bacteraemia leads to a higher rate of recurrence due to increased risk of new bacteraemia episodes added to treatment failures. Secondly, PWID are often in a poor socio-economic position and behavioural factors play a large role in recurrence [16].

Intravenous drug use is associated with a higher incidence of different types of infections [2, 17, 18]. *S. aureus* was the dominant microorganism in the first bacteraemia episodes (41.4%) and in the recurrent episodes (31.0%). *S. aureus* has been shown to be the most common microorganism among PWID [19–22]. PWID are also known to have a higher rate of nasal and skin colonisation of *S. aureus* [23]. The high prevalence of *S. aureus* found in our study is in contrast to the general Danish population, where *S. aureus* has been shown to account for 17% of all bacteraemia [7]. In our study there was only one case of *E. coli* bacteraemia – the most common pathogen in bacteraemia in the general Danish population [24]. However, this cohort consisted of relatively young patients whom injected illicit drugs through the skin where *S. aureus* is prominent [3, 4], whereas *E. coli* bacteraemia is more common in an elderly population and in connection with an infection focus in the urinary tract or abdomen [25]. Considering the low number of subjects in our study, we do not consider this finding to be of evidence that PWID have lower risk of *E. coli* bacteraemia than the general population. However, it indicates that *S. aureus* has a greater prevalence as causative agent in bacteraemia in this population. Simultaneously, there seems to be an increased risk of recurrence in particularly *S. aureus* bacteraemia. *S. pneumoniae* was the second most common microorganism of first bacteraemia episodes (10.3%), which supports a previous study showing that pneumonia is one of the most common infections among PWID [4]. The cohort had a high prevalence of co-infections, yet we found no significant association between co-infections and bacteraemia.

When studying predictive factors of bacteraemia, we found that psychiatric comorbidity was significantly associated with a lower risk of bacteraemia. This group did not differ from the rest of the cohort, except with regards to a lower risk of bacteraemia (data not shown). One explanation of this finding could be, that patients with psychiatric comorbidity in connection to their psychiatric treatment are more closely monitored. Hence, treatment for e.g. cutaneous abscesses would be initiated more promptly compared to other PWID.

Our study has some important limitations. The study design was retrospective and information regarding the cohort relies on previously recorded data: Firstly, not all hospital admissions related to intravenous drug abuse may not have been included in the study, since the primary screening in hospital database was done by using a limited set of search-terms. Secondly, data regarding gender, origin of infection, antibiotic and antiviral treatment and compliance were missing.

Nevertheless, the study has advantages; firstly, the personal identification number system used in Denmark that makes follow-up very easy and accurate; secondly, long study inclusion period (6 years) resulted in a larger number of cases with a long follow up period (10 years); thirdly, comprehensive questionnaires (from hospital journal database) and complete microbiological data were found for each patient.

## Conclusion

The incidence of bacteraemia and recurrent bacteraemia was high among hospitalised PWID and significantly associated with higher mortality. The most common microorganism of bacteraemia was *S. aureus,* which should be considered in empirical therapy, guided by local resistance-rates. Psychiatric comorbidity was significantly associated with a lower risk of bacteraemia, possibly due to a closer monitoring by the health care system of these patients.

## Supplementary information

**Additional file 1: Supplementary Figure 1.** Timeline for inclusion, gathering of microbiology data and follow up regarding hospitalised people who inject drugs with bacteraemia and recurrent bacteraemia.

**Additional file 2: Supplementary Table 1.** The HBV-, HCV- and HIV-status for hospitalised people who inject drugs.

**Additional file 3: Supplementary Table 2.** Blood culture isolates detected in hospitalised people who inject drugs.

## Data Availability

The datasets generated during the current study are not publicly available due to the sensitive personal data in a small cohort but are available from the corresponding author on reasonable request.

## Abbreviations

aOR: adjusted odds ratio
CI: Confidence interval
HBV: Hepatitis B
HCV: Hepatitis C
HIV: Human Immunodeficiency Virus
IV: Intravenous
OR: Odds ratio
OST: Opioid substitution treatment
PWID: People who inject drugs
