# Supplementary material for: Prevalence and recurrence of bacteraemia in hospitalised people who inject drugs – a single Centre retrospective cohort study in Denmark
Source: BMC Infect Dis. 2020 Aug 26;20:634. doi: 10.1186/s12879-020-05357-0 (PMC7448349; doi:10.1186/s12879-020-05357-0)
Supplement: Supplementary file 1 — Additional file 1: Supplementary Figure 1. Timeline for inclusion, gathering of microbiology data and follow up regarding hospitalised people who inject drugs with bacteraemia and recurrent bacteraemia. [file 12879_2020_5357_MOESM1_ESM.docx]

**Supplementary figure 1.** Timeline for inclusion, gathering of microbiology data and follow up regarding hospitalised people who inject drugs with bacteraemia and recurrent bacteraemia.

| Time | 2000 | |  |  |  |  |  | 2006 | | 2008 | | 2010 | |  |  |  |  |  | 2016 | |
| --- | --- | --- | --- | --- | --- | --- | --- | --- | --- | --- | --- | --- | --- | --- | --- | --- | --- | --- | --- | --- |
| (year) |  |  |  |  |  |  |  |  |  |  |  |  |  |  |  |  |  |  |  |  |
|  |  | Inclusion period | | | | | | |  |  |  |  |  |  |  |  |  |  |  |  |
|  |  |  |  |  |  |  |  |  |  |  |  |  |  |  |  |  |  |  |  |  |
|  |  | Microbiology data | | | | | | | | |  |  |  |  |  |  |  |  |  |  |
|  |  |  |  |  |  |  |  |  |  |  |  |  |  |  |  |  |  |  |  |  |
|  |  |  |  |  |  |  |  |  |  |  |  |  | Follow up | | | | | | |  |
|  |  |  |  |  |  |  |  |  |  |  |  |  |  |  |  |  |  |  |  |  |
