# Supplementary material for: Prevalence and recurrence of bacteraemia in hospitalised people who inject drugs – a single Centre retrospective cohort study in Denmark
Source: BMC Infect Dis. 2020 Aug 26;20:634. doi: 10.1186/s12879-020-05357-0 (PMC7448349; doi:10.1186/s12879-020-05357-0)
Supplement: Supplementary file 2 — Additional file 2: Supplementary Table 1. The HBV-, HCV- and HIV-status for hospitalised people who inject drugs. [file 12879_2020_5357_MOESM2_ESM.docx]

**Supplementary table 1.** The HBV-, HCV- and HIV-status for hospitalised people who inject drugs.

| **Viral infection** | | **Bacteraemia**  **(n = 58)**  **% (n)** | | **Non bacteraemia**  **(n = 199)**  **% (n)** | | **Bacteraemia with recurrence**  **(n = 29)**  **% (n)** | | **Non-recurrence bacteraemia**  **(n = 29)**  **% (n)** | |
| --- | --- | --- | --- | --- | --- | --- | --- | --- | --- |
| **HBV-status** | Acute/Chronic | 6.9 | (4) | 3.5 | (7) | 3.4 | (1) | 10.3 | (3) |
|  | Resolved/Immunised | 27.6 | (16) | 17.1 | (34) | 27.6 | (8) | 27.6 | (8) |
|  | Negative | 32.8 | (19) | 28.6 | (57) | 20.7 | (6) | 44.8 | (13) |
|  | Unknown | 32.8 | (19) | 49.2 | (98) | 48.3 | (14) | 17.2 | (5) |
| **HCV-status** | Acute | - | (0) | 0.5 | (1) | - | (0) | - | (0) |
|  | Chronic | 29.3 | (17) | 42.2 | (84) | 20.7 | (6) | 37.9 | (11) |
|  | Resolved | 5.2 | (3) | 4.5 | (9) | 10.3 | (3) | - | (0) |
|  | Chronic/Resolved | 29.3 | (17) | 20.6 | (41) | 27.6 | (8) | 31.0 | (9) |
|  | Negative | 1.7 | (1) | 3.5 | (7) | 3.4 | (1) | - | (0) |
|  | Unknown | 34.5 | (20) | 28.6 | (57) | 37.9 | (11) | 31.0 | (9) |
| **HIV-status** | Positive | 43.1 | (25) | 51.3 | (102) | 41.4 | (12) | 44.8 | (13) |
|  | Negative | 51.7 | (30) | 35.7 | (71) | 51.7 | (15) | 51.7 | (15) |
|  | Unknown | 5.2 | (3) | 13.1 | (26) | 6.9 | (2) | 3.4 | (1) |
| **HIV and HCV** | Co-infection | 27.6 | (16) | 36.2 | (72) | 24.1 | (7) | 31.0 | (9) |

If not stated otherwise, results are presented as n (%).

Abbreviations: PWID, people who inject drugs; HIV, Human Immunodeficiency Virus; HBV, hepatitis B; HCV, hepatitis C.
