# Supplementary material for: Prevalence and recurrence of bacteraemia in hospitalised people who inject drugs – a single Centre retrospective cohort study in Denmark
Source: BMC Infect Dis. 2020 Aug 26;20:634. doi: 10.1186/s12879-020-05357-0 (PMC7448349; doi:10.1186/s12879-020-05357-0)
Supplement: Supplementary file 3 — Additional file 3: Supplementary Table 2. Blood culture isolates detected in hospitalised people who inject drugs. [file 12879_2020_5357_MOESM3_ESM.docx]

**Supplementary table 2.** Blood culture isolates detected in hospitalised people who inject drugs.

| **Microorganism** | **All patients** | | **Patients with recurrent episodes** | | | |
| --- | --- | --- | --- | --- | --- | --- |
|  |  |  | **Findings in 1st episode** | | **Findings in 2nd episode** | |
|  | **%** | **(n)** | **%** | **(n)** | **%** | **(n)** |
| *Staphylococcus aureus* | 41.1 | (24) | 34.5 | (10) | 31.0 | (9^A^) |
| *Streptococcus pneumoniae* | 10.3 | (6) | 6.9 | (2) | 6.9 | (2) |
| *Streptococcus pyogenes* (Gr A) | 5.2 | (3) | - | (0) | - | (0) |
| *Streptococcus oralis* | 6.9 | (4) | 6.9 | (2) | - | (0) |
| Other non-hemolytic streptococci | - | (0) | - | (0) | 10.3 | (3) |
| Others | 36.2 | (21) | 51.7 | (15) | 51.7 | (15) |
| Total | 100 | (58) | 100 | (29) | 100 | (29^B^) |

^A^ 6/9 (67 %) with presumed same microorganisms as in the first episode.

^B^ 6/29 (21 %) with presumed same microorganisms as in the first episode.
